# Anaphylaxis: *Educational Programs Are Needed*

**DOI:** 10.1097/WOX.0b013e3181897d12

**Published:** 2008-10-15

**Authors:** Johannes Ring

**Affiliations:** 1 Department for Dermatology and Allergology, Technische Universität München, Munich, Germany

## 

Anaphylaxis is the number one emergency situation in allergy. More than 100 years after its first description, there are still many problems and open questions regarding pathophysiology, diagnosis, treatment, and prevention. Many national and international societies have edited guidelines that give clear recommendations for acute treatment, showing algorithms depending on the severity of the clinical symptomatology, which can be graded rather simply[1]. Adrenaline has been shown to be the most important drug to be applied, and the intramuscular route has a clear-cut advantage over other ways of administration in the initial phase. Recently, the World Allergy Organization has published a position statement on epinephrine application as a World Allergy Organization Report[2].

Although things seem pretty clear at a superficial glance, there are some controversies in detail with regard to when in the very early stage of anaphylaxis adrenaline should be given. There seems to be a divergence between pediatric allergists and allergists dealing more and mostly with adult patients because adrenaline has well-known pharmacological effects that can be detrimental to people with severe cardiac disease, especially arrhythmia. Furthermore, problems arise in prophylactic use, which means giving anti-anaphylactic treatment before development of symptoms after exposure to a known allergen. The situation is simple when it is a child who has inadvertently eaten peanuts and a history of severe peanut anaphylaxis.

The situation becomes more complex if a patient who had experienced an anaphylactic reaction in the past to an ubiquitous food such as cow's milk or hen's egg develops a feeling of uneasiness without clear-cut anaphylactic symptomatology and without knowing whether he has been exposed to the relevant allergen. This is even more important in patients in whom psychosomatic interaction plays a role and who may react in the direction of panic-fear attacks together with immunoglobulin E-mediated allergy. In these patients, coping with the anxiety is a major issue.

Although the use of adrenaline autoinjectors is simple and there is ample information with regard to correct application and a multitude of leaflets, it is surprising to see how often patients do not understand and--if they have understood--tend to forget the correct use of these devices. This phenomenon shows a surprising lack of relation to the intelligence quotient of the individual. Even doctors all of a sudden inject the adrenaline into their fingers when trying to handle the tools!

Therefore, in the management of patients with a history of anaphylaxis, it is crucial to communicate the information and the motivation to learn to the patients. This can best be achieved by structured educational programs. There is a long story of success concerning educational programs for asthma and eczema, where even controlled trials have been performed and proven the efficacy[3]. Similar programs have to be developed for anaphylaxis. In Germany, a working group named Anaphylaxis: Training and Education (acronym, AGATE) was founded recently that develops a structured educational program for "anaphylaxis schools," which will be studied in a controlled trial and hopefully implemented in Germany. Other countries are on the way to similar activities.

As old and historic as anaphylaxis may sound and as clear as pathophysiology and treatment may look in the textbooks, it remains a challenge for the patients and the practicing physician.

Johannes Ring

Editor-in-Chief

Munich, Germany, September 2008
